# On the Other Side of the Fence: Effects of Social Categorization and Spatial Grouping on Memory and Attention for Own-Race and Other-Race Faces

**DOI:** 10.1371/journal.pone.0105979

**Published:** 2014-09-02

**Authors:** Nadine Kloth, Susannah E. Shields, Gillian Rhodes

**Affiliations:** 1 Australian Research Council Centre of Excellence in Cognition and its Disorders, School of Psychology, The University of Western Australia, Crawley, Western Australia, Australia; 2 DFG Research Unit Person Perception, Friedrich Schiller University of Jena, Jena, Germany; Tel Aviv University, Israel

## Abstract

The term “own-race bias” refers to the phenomenon that humans are typically better at recognizing faces from their own than a different race. The perceptual expertise account assumes that our face perception system has adapted to the faces we are typically exposed to, equipping it poorly for the processing of other-race faces. Sociocognitive theories assume that other-race faces are initially categorized as out-group, decreasing motivation to individuate them. Supporting sociocognitive accounts, a recent study has reported improved recognition for other-race faces when these were categorized as belonging to the participants' in-group on a second social dimension, i.e., their university affiliation. Faces were studied in groups, containing both own-race and other-race faces, half of each labeled as in-group and out-group, respectively. When study faces were spatially grouped by race, participants showed a clear own-race bias. When faces were grouped by university affiliation, recognition of other-race faces from the social in-group was indistinguishable from own-race face recognition. The present study aimed at extending this singular finding to other races of faces and participants. Forty Asian and 40 European Australian participants studied Asian and European faces for a recognition test. Faces were presented in groups, containing an equal number of own-university and other-university Asian and European faces. Between participants, faces were grouped either according to race or university affiliation. Eye tracking was used to study the distribution of spatial attention to individual faces in the display. The race of the study faces significantly affected participants' memory, with better recognition of own-race than other-race faces. However, memory was unaffected by the university affiliation of the faces and by the criterion for their spatial grouping on the display. Eye tracking revealed strong looking biases towards both own-race and own-university faces. Results are discussed in light of the theoretical accounts of the own-race bias.

## Introduction

People are usually better at recognizing faces from their own than a different race, a phenomenon that is commonly referred to as the *own-race bias* ([1], for a review see [2], please note that in this paper, we use the term “race” to refer to visually distinct ethnic groups). Most attempts to explain the mechanisms that underlie this bias can be subsumed under two groups of theories, perceptual expertise accounts and sociocognitive accounts (for recent reviews and discussions of the two accounts, see [3]–[5]).

*Perceptual expertise accounts* claim that our face perception system has been shaped by our individual experience with faces, resulting in perceptual mechanisms and face representations that are ideally tailored to the faces that surround us (e.g., [6], [7]). Since most people have more exposure to faces from their own ethnic group than from other ethnic groups, their visual system is assumed to be tuned to distinguish between own-race faces better than other-race faces. Empirical support for the perceptual expertise account comes from studies demonstrating differences or delays in the perceptual processing of other-race faces relative to own-race faces (e.g., [8]–[14], for a recent review, see [15]). Importantly, such differences in perceptual processing seem to be reduced by differential expertise with other-race faces [16], [17]. Moreover, developmental studies have shown that the ORB is absent in very young children and only emerges during the first year of life [18], unless perceptual training with other-race faces during this critical stage prevents it ([19], for related findings, see [20]). Similarly, the ORB has been found to be absent [21] or even reversed [22] in Asian adults that were adopted and raised by European families, suggesting a strong influence of visual experience on our capacity to deal with faces from our own compared to other ethnicities.

*Sociocognitive accounts* of the ORB assume that the perceptual system would in principle be perfectly able to efficiently process faces from other ethnicities, but that reduced social interest in these faces leads to a more shallow processing compared to own-race faces. Possibly based on the early detection of a race-defining feature [23], [24], other-race faces are assumed to be initially categorized as belonging to a social out-group and, consequently, to be processed differently than own-race faces. Specifically, it has been suggested that the processing of other-race faces remains rather superficial and restricted to their group-defining features, leading to their categorization on a superordinate category level (e.g., as “Asian) rather than individuation [25].

Recent research has provided support for the idea that social in-group/out-group categorization can impact on memory for faces. Bernstein, Young, and Hugenberg [26] presented European American students with own-race faces, half of which were labeled as belonging to the participants' own university (social in-group categorization condition), the other half as belonging to a competing university (social out-group categorization condition). Social categorization substantially affected participants' memory for faces, with superior recognition of in-group relative to out-group faces (for similar findings, see [27]). This finding suggests that own-race bias-like memory biases can be induced even for faces of the participants' own race, for which perceptual processes should be optimized.

In a subsequent study, Shriver et al. [28] extended the investigation of effects of in-group/out-group-categorization to faces of a different race (African American) than the participants' (European American). Replicating Bernstein et al.'s [26] results, memory for faces of the participants' own race was impaired when these faces were introduced as belonging to a social out-group rather than the participants' in-group. However, memory for African American other-race faces was unaffected by social categorization and was always inferior to memory for own-race faces.

The finding that memory biases can be induced for own-race faces as a result of social categorization strongly suggests that social cognition and motivation can indeed affect face recognition, and might therefore contribute to the own-race bias. However, Shriver et al.'s [28] data suggest that memory for other-race faces cannot be improved as a result of social re-categorization as belonging to an in-group. This result is difficult to reconcile with the idea of social motivation and out-group classification as the main drivers of the own-race bias. Instead, the inability to improve recognition of other-race faces, even under conditions of increased social interest, is more in line with the idea that the perceptual system might just not be equipped to efficiently deal with other-race faces, as has been suggested by perceptual expertise accounts.

To account for the lack of enhanced recognition of other-race faces when these are categorized as in-group on a secondary social dimension, it has been suggested that the race of a face may be a category of such outstanding biological saliency that its processing is mandatory [28]. Consequently, effects of facial race might simply dominate any secondary social category information, and other-race faces might always be considered as out-group members and therefore not be readily individuated.

**Figure 1:**
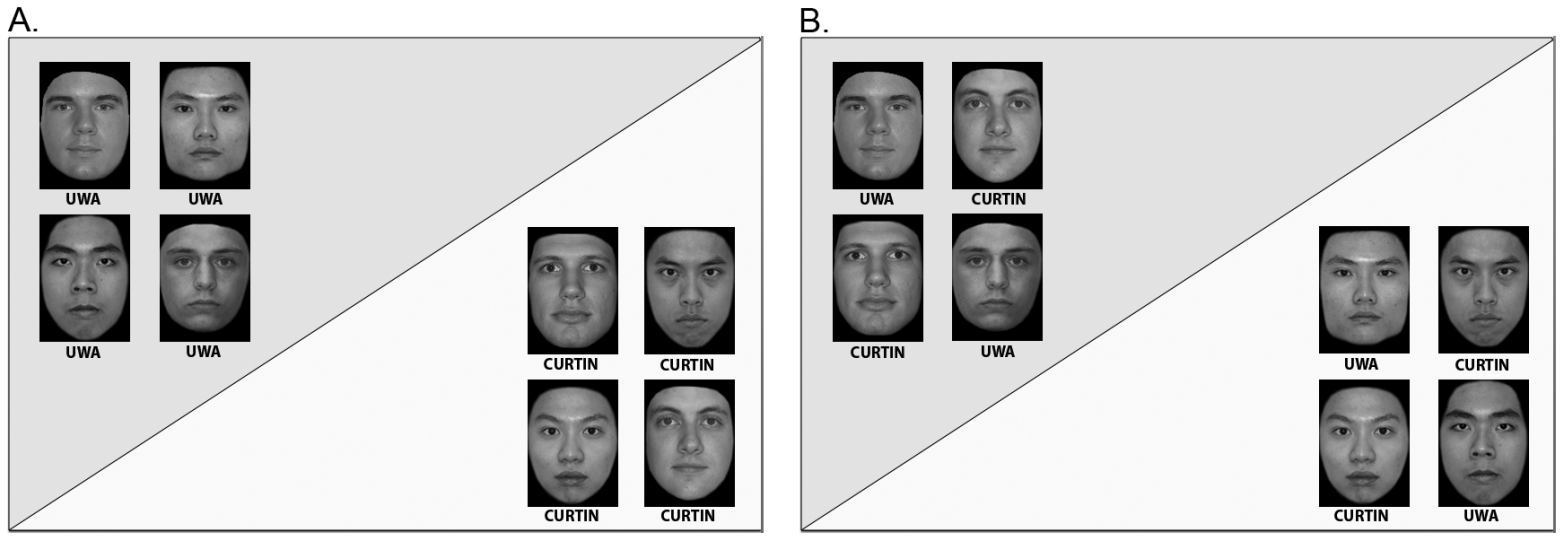
Examples of stimulus displays used during the study phase. A: Arrangement of faces in the university grouping condition. B: Arrangement of faces in the race grouping condition. The Asian faces depicted here were obtained from the Hong Kong Face Database, the Caucasian faces were obtained from Center for Vital Longevity Face Database. The authors obtained written permission from the owners of these databases to publish this figure under the CC-BY license.

Motivated by this reasoning, Hehman, Mania and Gaertner [29] developed a novel recognition memory paradigm in which the relative saliency of facial race and a secondary social categorization dimension (in this case university membership) could be manipulated. European American university students were asked to memorize the faces of European American and African American young adults. For each participant, half of the faces from each ethnic group were labeled as belonging to their social in-group, the other half as belonging to an out-group. Importantly, study faces were always presented in groups of eight with each group consisting of four European American and four African American individuals. Within each race, two faces were labeled as belonging to the same university as participants, the other two as belonging to a competing university. Varied between participants, faces were either spatially grouped according to their university affiliation, increasing the saliency of the university in-group/out-group dimension, or according to their ethnic background, increasing the saliency of the race in-group/out-group dimension (see Figure 1A and 1B in [29]). After studying five such displays containing a total of forty different faces, participants were presented with 80 individual test faces, consisting of the study faces as well as an equal number of new faces from each race that were randomly allocated university in-group and out-group labels. Participants' task was to indicate for each face whether it had been presented in the study phase (“old”) or not (“new”).

Hehman et al. [29] found evidence for an own-race bias only when faces were spatially grouped according to race. When faces were grouped according to university affiliation, the own-race bias was eliminated and participants showed an own-university bias instead, i.e., better recognition of faces from their own than the other university, irrespective of the race of the face.

Hehman et al.'s [29] paper represents an important milestone in the own-race bias literature since it is commonly interpreted as the first and only evidence for an eliminated own-race bias resulting from *increased* recognition performance for other-race faces that were categorized as belonging to the participants' university in-group, rather than decreased out-group recognition for own-race faces as reported by Shriver et al. [29]. Perceptual expertise accounts are typically thought of as being unable to explain such an improvement in the recognition of other-race faces up to the performance level for own-race faces. They assume a specialization of the perceptual system for own-race faces as a consequence of life-long exposure, which should not be easily overcome by means of re-categorization and/or a change in the spatial arrangement of the faces. Hehman et al.'s [29] data therefore provide the strongest support for the sociocognitive account of the own-race bias to date.

The main aim of the present study was to establish whether the currently singular findings of Hehman et al. [29] are robust in the sense that they extend to participants and faces from other cultural and ethnic backgrounds. Hehman et al.'s observations are solely based on participants from one ethnic background, European Americans, studying European American and African American faces. While obvious practical reasons make restricting participants to only one ethnic group a common procedure in the own-race bias literature (for other examples, see [26], [28], [30], [31]), collecting data from both ethnicities that are used as stimulus faces generally controls for any stimulus effects and allows to get a more thorough understanding of the generalizability of any observed effects across different face and participant ethnicities. For this reason, we decided to test participants from two ethnic backgrounds: an Australian population of European descent and a population of Asian students that only recently migrated to Australia. Participants were asked to memorize Asian and European Australian faces that were presented in two spatially separated groups of four. Half of the faces from each race were labeled as belonging to the participants' own or a competing university, respectively. Between participants, individual faces were allocated to the two groups on the screen based either on their race or their university affiliation. Given Hehman et al.'s results, we expected that the effect of face race (own-race, other-race) on participants' memory for faces would be qualified by an interaction with the university affiliation condition (own-university, other-university) and the criterion for the spatial grouping of the faces (grouped by race, grouped by university affiliation), reflecting the absence of an own-race bias when faces are classified as belonging to the participants' own university and are spatially grouped according to their university affiliation rather than race.

An additional aim of our study was to explore potential effects of the faces' university affiliation and the spatial grouping criterion on participants' distribution of overt attention towards the faces during the study phase. To this end, we employed eye tracking to establish whether systematic biases in looking behavior were associated with the predicted effects of university affiliation and the spatial grouping criterion on the own-race bias. For instance, Hehman et al.'s [29] results would be in line with the idea that participants' attention is generally biased towards the own-group half of the screen, whether the faces are grouped according to their university affiliation or their race. If this were the case, we would expect participants in the race grouping condition to be spending more time looking at own-race than other-race faces, irrespective of university affiliation. Participants in the university grouping condition would be expected to be spending more time looking at own-university than other-university faces, irrespective of race. Consequently, own-university other-race faces would be attended more in the university grouping than in the race grouping condition.

## Methods

### Ethics statement

This research was in accordance with the Declaration of Helsinki and approved by the Human Research Ethics Committee of the University of Western Australia. Data was anonymized at collection. Each dataset was saved under a unique alphanumeric code that did not contain information about the identity of the participant. Written informed consent was obtained from all participants before the experiment, in accordance with the National Statement on Ethical Conduct in Human Research of the Australian Government National Health and Medical Research Council and the Australian Research Council. The informed consent was signed with the participants' full name and dated from the day of data collection. Therefore, we did obtain identifying information that, via the dates, the authors could potentially relate to the datasets. However, consent forms are archived separately from the experimental data sets.

### Participants

Forty Asian (12 male, 18–30 years, *M* = 20.8, *SD* = 2.3) and forty Australian undergraduate students of European descent (henceforth “European”, 15 male, 17–56 years, *M* = 20.1, SD = 6.3) from the University of Western Australia participated. On average, Asian participants had lived in Australia for 2.5 years (*SD* = 1.5, range: 1 to 6 years). All participants had normal or corrected-to-normal vision and were naïve to the purpose of the study. Participants received course credit or a monetary reward of 5 AUD for their participation.

### Stimuli

Pictures of 80 unfamiliar young male faces (40 Asian, 40 European Australians) with direct gaze and neutral emotional expression were used as stimuli. Sixty faces (30 per ethnic group) had been used in previous research [32], and twenty additional faces (10 per ethnic group) were sourced from the Center for Vital Longevity Face Database (http://agingmind.utdallas.edu/stimuli/facedb/, [33]) and from the internet.

Faces were converted into black-and-white images and pasted onto a uniform black background. To minimize external cues for recognition, a black mask occluded as much of the hair as possible. None of the faces had beards, tattoos or piercings. Study faces were resized to a height of 5 cm and a width of 4.3 cm, corresponding to 5.5°×4.7° at a viewing distance of 52 cm. The label “UWA” (short for the University of Western Australia) or “Curtin” (another university in the same city) was placed underneath each face to indicate its association with one or the other university. The assignment of individuals to universities was counterbalanced across participants and none of the faces actually currently attended either university.

During the study phase, participants were asked to memorize 40 faces that were presented on five consecutive displays each containing eight faces. Within a single display, faces were separated into two groups of four individuals each. Depending on the experimental condition, faces were either grouped according to their race or according to their university affiliation. Groups were positioned on opposite halves of a rectangle, with a black diagonal clearly separating the two halves. To additionally enhance perceptual cues to grouping, the background colour of the two halves differed slightly (Figure 1).

The diagonal dividing the two halves of the rectangle could go from the top left to the bottom right or from the bottom left to the top right. The assignment of own-university or own-race groups to the triangles of the display was not predictable for each participant and was fully counterbalanced across participants, as was the order of the Asian and European faces within the university sub-groups and the UWA and Curtin faces within the race sub-groups. Specifically, individual Asian or European faces or faces labeled “UWA” and “Curtin” were equally likely to appear on the top-left/bottom-right or top-right/bottom-left location within their group of four faces.

During the test phase of the experiment, faces were presented individually at a size of 7.5 by 6.1 cm, corresponding to 8.3° by 6.7°. The forty faces that had not been presented during learning were randomly assigned university labels, making sure there were an equal number of new faces labeled “UWA” and “Curtin” for each race.

### Apparatus

Stimuli were presented on a matte 27-inch iMac LED screen with a resolution of 1920×1080. The experimental procedure was programmed using SR Research Experiment Builder. A standard computer keyboard was used to record participants' responses.

During the learning phase of the experiment, participants' eye movements were recorded using a remote SR Research EyeLink 1000 eye tracking system with a sampling rate of 1000 Hz. A chin-rest ensured minimal head movements and a constant viewing distance of 52 cm.

### Procedure

The experiment consisted of an initial learning phase followed by a recognition test. During the learning phase participants were presented with five displays that each contained four Asian and four European faces, presented in two separate groups of four. Depending on the experimental condition, faces were either grouped according to their race or their university affiliation (Figure 1). Each learning display remained on the screen for 16 seconds, allowing for an average encoding time of 2 seconds for each face. There was an inter-trial interval of 500 ms between learning displays.

In the recognition test, participants were presented with 80 individual test faces, 40 of which they had already seen in the learning phase. Faces were presented in randomized order. The task was to indicate for each face whether it was old or new, by pressing one of two labeled keys. Faces stayed on the screen until a response was made. Old faces were presented with the same university labels as in the learning phase and new faces were randomly assigned university labels, making sure there were an equal number of new faces labeled “UWA” and “Curtin” for each race. Across participants, each individual face was presented with each university affiliation equally often and was equally likely to be an “old” face, i.e., presented in both study and recognition phase, or “new”, i.e., presented only in the recognition phase.

To familiarize participants with the layout of the stimuli and the timing of the presentation, five demonstration displays using cartoon characters from the TV show “The Simpsons” preceded the learning phase. The recognition test was also preceded by the presentation of four example stimuli to familiarize participants with the size and location of the test stimuli. These example stimuli showed two Asian and two European faces, one of each labeled “UWA” and “Curtin”, respectively. The identities shown on these example stimuli were not part of the stimulus set for the experiment proper.

To ensure accurate measurement of participants' eye movements, the experiment was preceded by a nine-point calibration and validation procedure of the eye tracker. Calibration was repeated if the maximum error was larger than 1°. An additional drift check was performed at the center of the screen after each individual learning display to ensure accurate measurements of eye movements throughout the learning phase. If the drift check indicated a loss in tracking accuracy, the full nine-point calibration procedure was repeated before the next learning display was presented.

## Results

Signal detection measures for sensitivity of recognition memory (d′) and response bias (C) were calculated separately for each condition. Hit and false alarm rates of zero or one were adjusted to 0.5/n or (n – 0.5)/n, respectively, with n being the possible number of hits or false alarms [34].

### Recognition memory sensitivity (d′)

A repeated measures analysis of variance (ANOVA), with Face race (own-race, other-race) and Face affiliation (own-university, other-university) as within-participants factors and Participant race (Asian, European) and Grouping condition (grouped by race, grouped by university) as between-participants factors was conducted on d′ scores.

**Figure 2:**
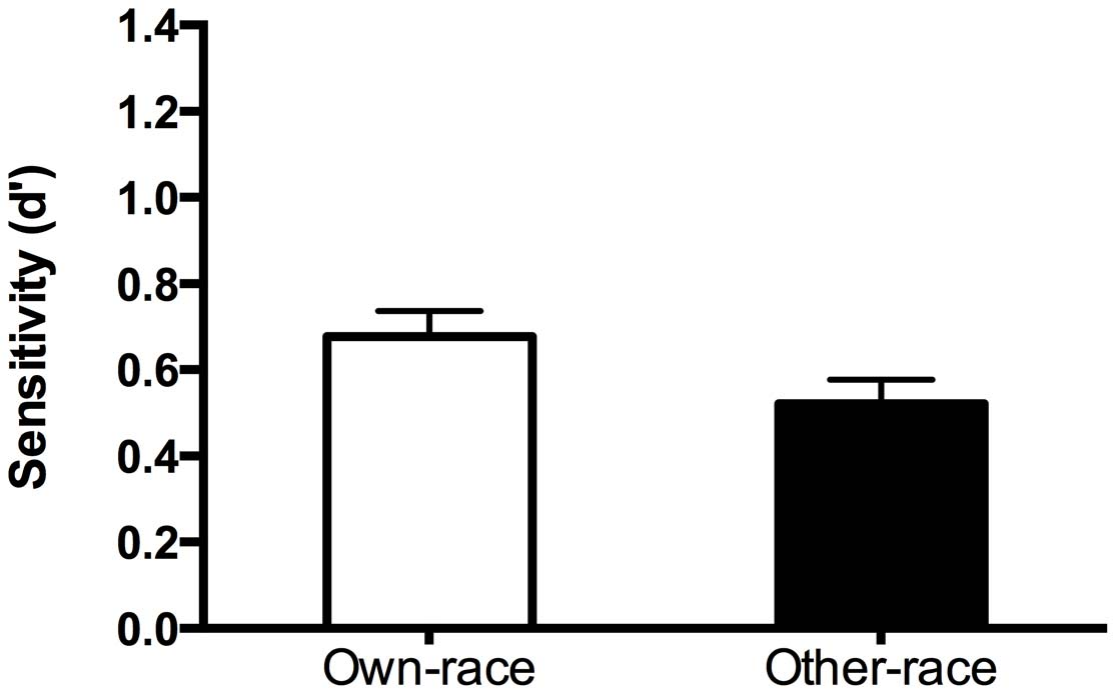
Mean d′ for own-race and other-race faces. Error bars show standard errors of the mean.

The analysis revealed a significant main effect of Face race, *F*(1, 76) = 4.15, *p* = .045, η_p_^2^ = .052 (Figure 2), reflecting better recognition for faces of the participants' own race (*M* = 0.68, *SEM* = 0.06) than for faces of the other race (*M* = 0.52, *SEM* = 0.06).

**Figure 3:**
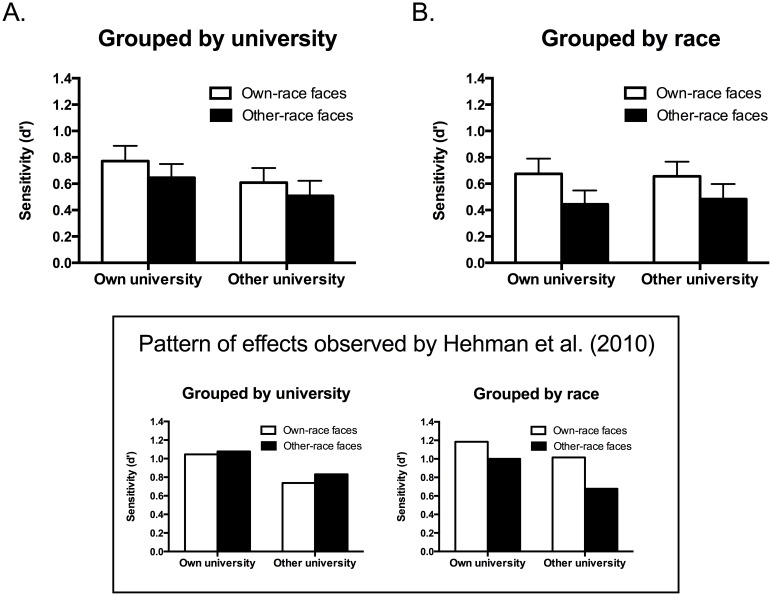
A. Mean d′ for own-race and other-race faces in the university grouping condition. B. Mean d′ for own-race and other-race faces in the race grouping condition. Error bars show standard errors of the mean. Box: Pattern of effects observed by Hehman et al. [29].

Importantly, the predicted interaction of Face race x Face affiliation x Grouping condition was not significant, *F*(1, 76) = 0.14, *p* = .907, η_p_^2^ = .000 (Figure 3), indicating that the own-race bias was not eliminated when faces were grouped by university. No other effects were significant, including the main effects of Face affiliation, *F*(1, 76) = 0.70, *p* = .41, η_p_^2^ = .009, Participant race, *F*(1, 76) = 1.18, *p* = .28, η_p_^2^ = .015, and Grouping Condition, *F*(1, 76) = 0.64, *p* = .43, η_p_^2^ = .008, (all other *F*s<1.12, all *p*s>.29, all η_p_^2^<.016).

Despite the absence of the predicted interaction of Face race, Face affiliation and Grouping condition, visual inspection of Figure 3 might be taken to suggest that the own-race memory bias observed in the grouped by university condition is somewhat attenuated relative to the bias in the grouped by race condition. We therefore conducted two additional one-way between-participants ANOVAs to directly compare the effect of grouping condition on the magnitude of the own-race memory bias (d′ for own-race faces - d′ for other-race faces) for own-university and other-university faces. In line with the results of the overall ANOVA, there was no significant difference in the magnitude of the bias between grouping conditions for faces in the own-university condition, *F*(1, 78) = 0.21, *p* = .65, η_p_^2^ = .002, or the other-university condition, *F*(1, 78) = 0.17, *p* = .68, η_p_^2^ = .002.

### Response bias (C)

**Figure 4:**
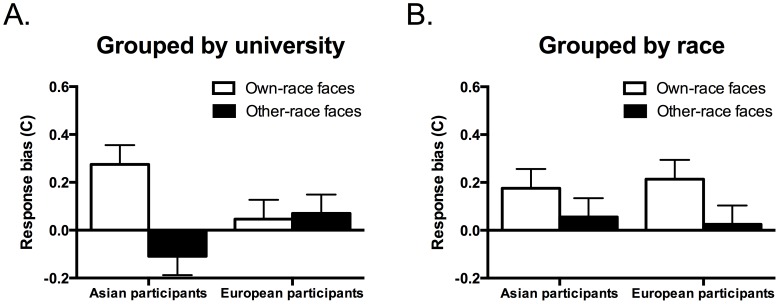
A. Mean response bias (C) for own-race and other-race faces in the university grouping condition. B. Mean response bias (C) for own-race and other-race faces in the race grouping condition. Error bars show standard errors of the mean.

The same ANOVA was conducted on response bias (C) scores. There was a significant main effect of Face race, *F*(1, 76) = 11.17, *p* = .001, η_p_^2^ = .13, indicating a more conservative response bias, i.e., a greater reluctance to classify faces as old, for own-race (*M* = 0.18, *SEM* = 0.04) than other race faces (*M* = 0.01, *SEM* = 0.04). This main effect was qualified by a three-way interaction of Face race, Participant race, and Grouping condition, *F*(1, 76) = 5.66, *p* = .02, η_p_^2^ = .07 (Figure 4).

To follow up on the three-way interaction, separate ANOVAs were run for the university grouping and the race grouping conditions. For the university grouping condition, there was a main effect of Face race, *F*(1, 38) = 7.12, *p* = .011, η_p_^2^ = .16, and an interaction of Face race with Participant race, *F*(1, 38) = 9.11, *p* = .005, η_p_^2^ = .19. Asian participants had a significantly larger response bias for own-race faces than other-race faces, *t*(19) = 4.15, *p* = .001, d = 1.06, whereas European participants did not, *t*(19) = .241, *p* = .812, d = 0.07 (Figure 4). Additional one-sample *t* tests revealed that Asian participants had a conservative response bias that was significantly different from zero for own-race faces, *t*(19) = 3.34, *p* = .003, d = 0.75, but not for other-race faces, *t*(19) = −1.38, *p* = .184, d = −0.31, whereas European participants had no significant response biases towards either own-race faces, *t*(19) = 0.62, *p* = .54, d = 0.14, or other-race faces, *t*(19) = 0.85, *p* = .41, d = 0.19, (Figure 4).

The ANOVA for the race grouping condition revealed a significant main effect of Face race, *F*(1, 38) = 4.37, *p* = .043, η_p_^2^ = .10, indicating a more conservative bias for own-race faces (*M* = 0.20, *SEM* = .06) than other-race faces (*M* = 0.04, *SEM* = .05). One-sample *t* tests confirmed that only the bias for own-race faces was significantly different from zero, *t*(39) = 3.36, *p* = .002, d = 0.53, whereas there was no significant bias for other-race faces, *t*(39) = 0.75, *p* = .457, d = 0.12. There were no other significant effects, including the main effects of Face affiliation, *F*(1, 76) = 1.74, *p* = .19, η_p_^2^ = .022, Participant race, *F*(1, 76) = 0.03, *p* = .87, η_p_^2^ = .000, and Grouping Condition, *F*(1, 76) = 0.57, *p* = .45, η_p_^2^ = .007, (all other *F*s<2.9, *ps*>.095, η_p_^2^<.069).

### Eye tracking data

The dependent variable was the mean proportion of viewing time during the learning phase spent looking at individual faces (face plus university label) within the display (Table 1). A repeated measures ANOVA was conducted with Face race (own-race, other-race) and Face affiliation (own-university, other-university) as within-participants factors and Participant race (Asian, European) and Grouping condition (grouped by race, grouped by affiliation) as between-participants factors.

**Table 1:** Proportion of viewing time spent looking at own-race and other-race faces affiliated with the participants' own or another university for Asian and European participants in the race and university grouping conditions.

Face race	Univ.	Asian participants (N = 40)				European participants (N = 40)			
		Grouped by race		Grouped by university		Grouped by race		Grouped by university	
		*M*	*SEM*	*M*	*SEM*	*M*	*SEM*	*M*	*SEM*
Own- race	**Own**	0.277	0.013	0.277	0.012	0.245	0.009	0.259	0.009
	**Other**	0.227	0.013	0.215	0.012	0.237	0.007	0.261	0.007
	**Total**	**0.504**	**0.008**	**0.492**	**0.007**	**0.483**	**0.013**	**0.520**	**0.012**
Other- race	**Own**	0.250	0.015	0.266	0.014	0.244	0.008	0.238	0.008
	**Other**	0.206	0.015	0.204	0.012	0.242	0.009	0.217	0.010
	**Total**	**0.456**	**0.009**	**0.470**	**0.009**	**0.487**	**0.013**	**0.455**	**0.011**

Please note that the proportions do not add up exactly to one, since some proportion of the viewing time was spent on saccades and fixations outside of interest areas.

There was a significant main effect of Face race, *F*(1, 76) = 10.06, *p* = .002, η_p_^2^ = .12. Participants spent longer viewing own-race (*M* = .500, *SEM* = .005) than other-race faces (*M* = .467, *SEM* = .005). There was also a significant main effect of Face affiliation, *F*(1, 76) = 10.99, *p* = .001, η_p_^2^ = .13, reflecting a looking bias towards own-university faces (*M* = .514, *SEM* = .010) over other-university faces (*M* = .452, *SEM* = .009). Both main effects were further qualified by higher order interactions: There was a two-way interaction of Face affiliation and Participant race, *F*(1, 76) = 6.38, *p* = .014, η_p_^2^ = .077, and a three-way interaction of Face race, Participant race, and Grouping Condition, *F*(1, 76) = 5.31, *p* = .024, η_p_^2^ = .07. No other effects were significant (all *F*s<1.63, all *p*s>.20, all η_p_^2^<.03).

**Figure 5:**
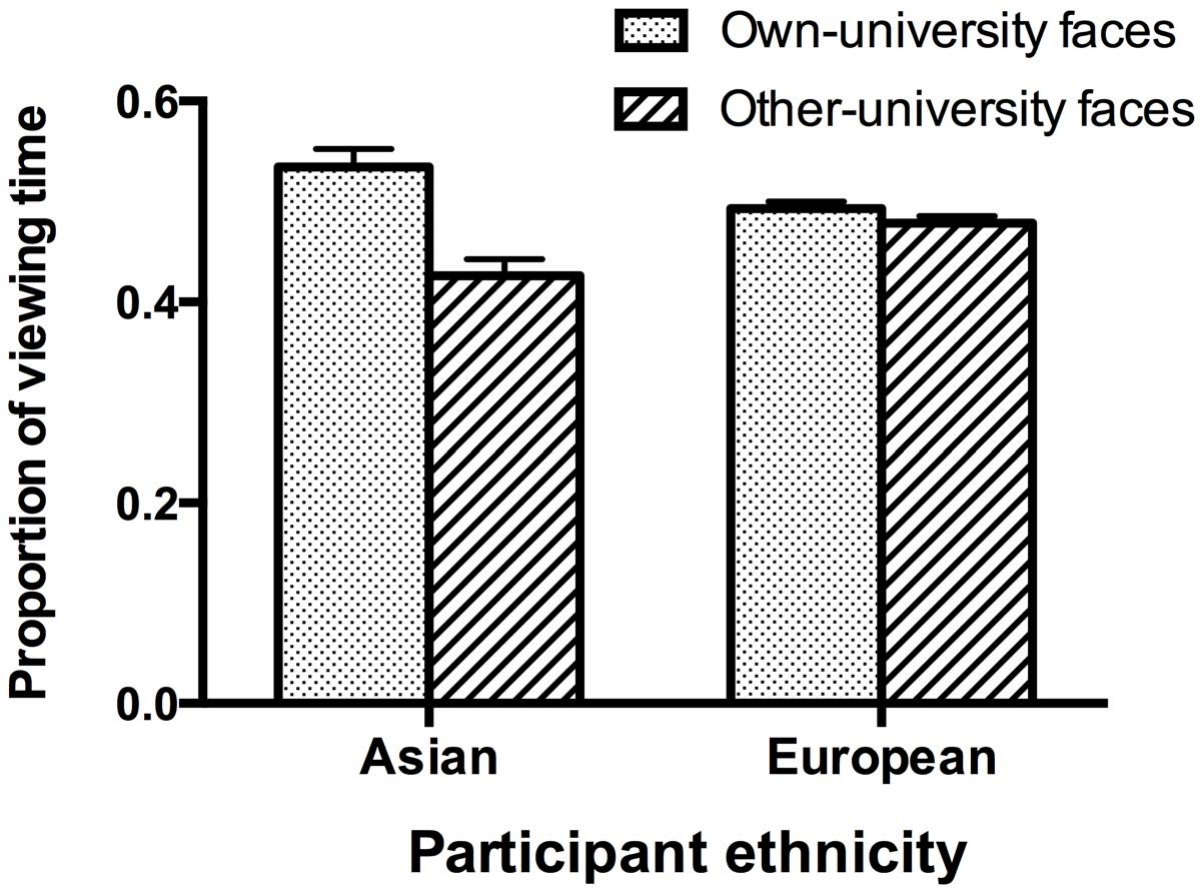
Mean proportion of viewing time spent looking at own-university and other-university faces for Asian and European participants. Error bars indicate standard errors of the mean.

Bonferroni-corrected *t* tests were conducted to further explore the Face affiliation x Participant race interaction (Figure 5). They revealed that Asian participants had a significantly greater looking bias towards faces that were labeled own-university (*M* = .535, *SEM* = .017) compared to other-university faces (*M* = .426, *SEM* = .017), *t*(39) = 3.19, *p* = .009, d = 1.01. A numerical trend in the same direction in European participants was not significant, *t*(39) = 1.05, *p* = .302, d = 0.33 (Figure 5).

**Figure 6:**
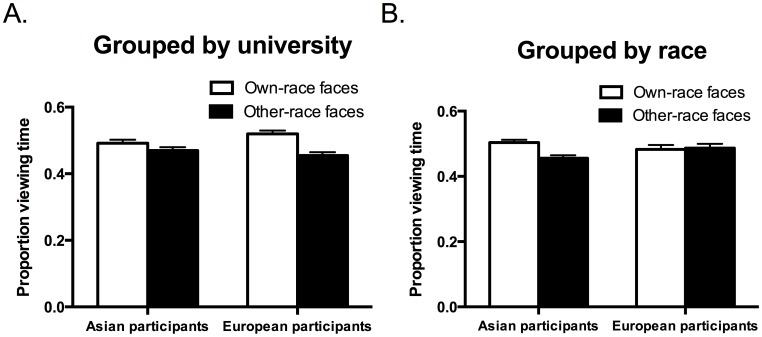
A. Mean proportion of viewing time spent on own-race and other-race faces for Asian and European participants in the university grouping condition. B. Mean proportion of viewing time spent on own-race and other-race faces for Asian and European participants in the race grouping condition. Error bars indicate standard errors of the mean.

The interaction of Face race, Participant race, and Grouping Condition (Figure 6) was followed up with separate ANOVAs for each grouping condition. In the university grouping condition, there was a main effect of Face race, *F*(1, 38) = 9.89, *p* = .003, η_p_^2^ = .21, reflecting a looking bias towards own-race faces (*M* = .506, SEM = .007) over other-race faces (*M* = .462, *SEM* = .007). No other effects were significant, *F*<2.4, *p* = .13, η_p_^2^ = .06.

The ANOVA for the race grouping condition revealed a trend towards an interaction of face race with participant race, *F*(1, 38) = 2.92, *p* = .096, η_p_^2^ = .071. Asian participants spent a larger proportion of their viewing time looking at own-race faces (*M* = .504, *SEM* = .008) than other-race faces (*M* = .456, SEM = .009), *t*(19) = 2.87, *p* = .01, d = 1.26. European participants did not show such a bias to look at own-race faces (*M* = .483, *SEM* = .013) over other-race faces (*M* = .487, SEM = .013), *t*(19) = 0.16, *p* = .874, d = 0.07. No other effects were significant, *F*<2.1, *p*>.15, η_p_^2^ = .05.

Finally, correlational analyses were run to directly examine the relationship between the looking bias and the memory bias for own-race faces. To measure the magnitude of the attentional bias towards own-race faces we subtracted the proportion of dwell time spent on other-race faces from the proportion of dwell time spent on own-race faces (*M* = .033, *SEM* = .011). This relative dwell-time measure was correlated with the difference in d's for own-race and other-race faces (*M* = .157, *SD* = .076). There was no significant correlation between these two measures, *r*(78) = .07, *p* = .54.

Interestingly, there was a significant correlation between the looking bias (*M* = .062, *SEM* = .019) and the memory bias (*M* = .07, *SEM* = .083) for own-university faces, *r*(78) = .284, *p* = .011. In summary, the correlational analyses indicated that while there was no relationship between the looking bias and the memory bias for own-race over other-race faces, those participants with a stronger looking bias for own-university over other-university faces also showed a stronger memory bias in favor of own-university faces.

## Discussion

Participants had better memory for faces from their own ethnic group than for faces from another ethnic group. Importantly, the memory bias for own-race faces was completely unaffected by either the university affiliation of the faces or by the nature of their spatial grouping (by university or by race). We therefore did not replicate Hehman et al.'s [29] finding that categorizing faces as belonging to the participants' own university eliminated the own-race bias when faces were spatially grouped according to university affiliation rather than race.

In addition to the memory bias for own-race faces, eye tracking revealed that participants were also biased to spend more time *looking* at own-race than other-race faces (with the exception of Caucasian participants in the race grouping condition). This attentional bias indicates an increased interest in own-race faces, which is an interesting finding considering that sociocognitive accounts have suggested differences in social interest for own-race and other-race faces to be amongst the main drivers of the ORB (e.g., [35]). However, we found no correlation between this attentional bias and the memory bias for own-race faces. Participants with stronger attentional biases towards own-race faces did not also exhibit larger own-race biases in memory. Our data therefore do not support the strong relationship between differential social interest for own-race and other-race faces and differential memory performance for the two ethnic groups that has been suggested by sociocognitive accounts.

A possible reason why we did not replicate Hehman et al.'s [29] findings is related to the substantial differences in the editing of the stimulus faces between the two studies. The stimuli we used in our study showed only the actual faces in front of a standardized black background and were edited to be perfectly upright and to exclude hair or other potential external cues to recognition. In contrast, the stimuli used by Hehman et al. were more heterogeneous (cf., Figure 1 in [29]). First and foremost, hairstyles were visible. Furthermore, stimuli varied with respect to the posture/tilt of the heads and the color of the background. Finally, parts of the clothing were visible around the neckline in some of Hehman et al.'s stimuli. Any of these sources of variation might have introduced potential memory cues in addition to the identity and ethnicity information contained in the stimulus faces. The presence of such external cues might have helped participants to improve their memory performance for individual images. Critically, some or even all of this improvement might have happened without participants even having to make use of face processing mechanisms. Under such circumstances, an improvement of other-race face recognition as a result of in-group categorization, even up to the performance level of own-race faces, is not at all inconsistent with the perceptual expertise model.

The idea that these differences in inter-stimulus variance and stimulus memorability between the two studies might be powerful enough to explain differences in results receives some support from the fact that the recognition memory performance of our participants was generally lower than that of Hehman et al.'s [29] participants. It is plausible that this general performance decrease is due to the absence of extra-facial memory cues in our stimulus set, which made it more challenging for our participants to perform well on the memory test, and also provided less potential strategies to improve recognition performance as a result of increased motivation for other-race faces affiliated with the participants' own university. Potentially related to the differences in overall recognition memory performance between studies, it is important to acknowledge that relative to Hehman et al.'s study, the effect size of the own-race bias observed in our study was considerably smaller. Although our statistical analysis only revealed a main effect of face race on participants' recognition memory, it is difficult to completely rule out that our small effect size might have made it more difficult to detect a reliable reduction in the own-race bias due to the different grouping conditions.

A second potential explanation for the fact that we did not replicate Hehman et al.'s [29] effects on the ORB might be that cultural differences led to a differential effectiveness of the social categorization manipulation for the two samples. Following Hehman et al., we chose university affiliation as the secondary social categorization dimension. However, the feeling of belonging to one's own university, leading to enhanced in-group identification, and rivalries between different universities, resulting in strong out-group categorizations, might be less pronounced in Australia than in the United States of America [36]. The lack of sports competitions between Australian universities, the absence of fraternities and sororities, and the very small percentage of students moving cities to go to university and living on campus might be factors that contribute to Australian students identifying themselves less with their university than American students.

Nevertheless, it would be too simplistic to merely discard the university affiliation manipulation as ineffective for our participants. We found that categorizing faces as belonging to their social in-group or out-group significantly affected participants' looking behavior. Specifically, Asian participants devoted more of their limited viewing time to those faces that were labeled as belonging to their own university than to faces that were labeled as belonging to the other university. This finding suggests that the social categorization manipulation effectively affected participants' motivation to explore the different faces of the screen, biasing them to overtly attend to faces from their university in-group more than faces from a university out-group. European participants showed the same tendency to attend to own-university faces more than other-university faces, but their looking bias for own-university faces was much smaller than that of Asian participants and was not significant.

Interestingly, participants who were more strongly biased to attend to own-university faces over other-university faces also showed larger memory biases in favor of own-university faces (for related findings, see [37]). This finding might indicate that the direct relationship between enhanced social interest and superior memory performance that has been suggested by the sociocognitive account might only exist for in-group/out-group constellations on social dimensions other than ethnicity. In contrast, face ethnicity might be an in-group/out-group dimension for which limited perceptual experience and inefficient perceptual strategies impose limitations on the extent to which processing efficiency and memory can be improved for out-group faces (cf., [3]).

In addition to the looking behavior, the spatial grouping of faces also affected participants' response criterion (C). In signal detection theory, the response criterion (C) is conceived as a measure for general response biases or heuristics [38]. It is a common finding in the own-race bias literature that participants respond more conservatively to own-race than other-race faces, that is, they show a greater reluctance to classify own-race faces as having been presented during the study phase [2]. In our study, Asian participants in both grouping conditions as well as European participants in the race grouping condition showed these conservative biases for own-race faces. Grouping faces according to university affiliation, rather than race, eliminated this conservative response bias, at least in European participants. This pattern of results suggests that spatial grouping was capable of affecting other-race face processing to some extent. However, it did not affect the own-race bias at a level of perceptual sensitivity, but rather at the level of a decision criterion.

A general difficulty in the interpretation of research on the ORB is imposed by the fact that the perceptual expertise account and the sociocognitive account are not as selective or clear-cut as they might appear at first description. There is some overlap between the accounts and some findings typically interpreted as supporting one model are actually not explicitly ruled out in the other. For instance, the finding that categorization of own-race faces as belonging to a social out-group can reduce recognition memory performance and induce in-group/out-group memory biases for faces of one's own race [26], [28] is typically interpreted as evidence for the sociocognitive account and, at least implicitly, as evidence against the perceptual expertise account. However, the perceptual expertise account does not claim that motivational or attentional factors do not play any role in face recognition memory (for a recent discussion of this aspect, see [3]). Obviously, many perceptual processes will benefit from increased levels of motivation or attention and, therefore, differential motivation to identify faces from different groups can lead to differences in recognition performance. Where the perceptual expertise account clearly differs from sociocognitive approaches, however, is in the prediction that under equal, even optimal, conditions of motivation and attention to individuate own-race and other-race faces, participants will be less *able* to individuate and recognize own-race faces than other-race faces (unless they had substantial perceptual expertise with faces from the other ethnicity). The only study to date whose findings are interpreted to be clearly not in line with this assumption is the one by Hehman et al. [29] whose findings we were unable to replicate here.

In this context, we would also like to point out that despite commonly being interpreted as evidence for an elimination of the own-race bias “by increasing recognition for other-race faces” ([29], p. 447), Hehman et al. [29] do not actually report a direct comparison of the recognition performance for own-university other-race faces in the university-grouping and the race-grouping conditions. Visual inspection of the data (cf., Figure 2 in [29]) suggests that the recognition performance for own-university other-race faces is almost identical in the two spatial grouping conditions. Instead, what seems to underlie the presence of an ORB in the race grouping condition but not in the university grouping condition is a better recognition performance for own-university own-race faces in the race grouping than in the university grouping condition (cf., Figure 2 in [29]).

Given the difficulties of both perceptual expertise and sociocognitive models to fully explain the origin of the own-race bias on their own, the emergence of more integrated accounts is a promising development. For instance, the categorization-individuation model (CIM) suggests an interaction of perceptual, attentional, and motivational drivers to underlie the own-race bias [35], [39]. This model assumes that a face automatically activates category information, which in turn directs the observers' attention to category-defining facial features. Since category activation is assumed to be stronger for other-race faces, these faces are likely to appear more homogeneous as a class than own-race faces and so are more easily misidentified. The CIM assumes that such homogeneity effects can potentially also occur for own-race faces, and predicts that these will appear more homogeneous after category activation. Categories are assumed to direct participants' attention, such that own-race faces or, more generally, in-group categories signal the importance of individuating a person, whereas other-race faces or, more generally, out-group categories signal the irrelevance of identity. Moreover, the model assumes that situational cues can redirect selective attention to the identity of other-race faces, which might influence the own-race bias, particularly for perceivers with a high level of individuation experience with other-race faces. Their experience is assumed to provide them with the necessary perceptual efficiency to process facial information that best distinguishes between individuals. Crucially, the model assumes that observers may not fully use these individuation capacities unless they are motivated to do so and that differential experience with own-race and other-race faces “operates in concert with differential motives to individuate” ([35], p. 1171).

Importantly, if several different factors potentially underlie the own-race bias, their relative contributions may differ between individuals and possibly also between different social contexts and demographic compositions. Consequently, the own-race bias observed in North American participants studying European American and African American faces might be more susceptible to a potential influence of categorization on a secondary social dimension than biases observed in other contexts. Tentative support for this idea might come from a recent study that found that effects of in-group categorization of other-race faces on the magnitude of the ORB depended on participants' expectations of the relative probability of future contact with in-group and out-group members [40].

Overall, our study showed that while categorizing faces as belonging to their own or a different university biased participants' eye movements towards in-group faces, and the criterion for spatial grouping affected their response criterion as well as their eye movements, it was only the actual race of the study faces that had the power to affect face recognition memory. Participants in our study showed a clear own-race bias that was unaffected by either the perceived university affiliation of the faces or by the nature of the spatial grouping of the faces. Our data therefore provide no evidence that social categorization alone can affect memory for own-race and other-race faces to the extent that the own-race bias is eliminated. Our finding is more in line with the idea that differential perceptual expertise with own-race and other-race faces is a critical component of the own-race bias.
